# Advancing Intracranial Tumor Treatment Through Gyroscopic Radiosurgery-Based Lattice Therapy: Evidence From a Case Series Study

**DOI:** 10.7759/cureus.85383

**Published:** 2025-06-05

**Authors:** Melek Tugce Yilmaz, Fazli Yagiz Yedekci, Sepideh Mohammadipour, Huseyin Kivanc, Mustafa Cengiz, Gokcen Cifci, Neyran Kertmen, Gozde Yazici

**Affiliations:** 1 Radiation Oncology, Hacettepe University Medical School, Ankara, TUR; 2 Radiation Oncology, Hacettepe University, Ankara, TUR; 3 Radiology, Hacettepe University, Ankara, TUR; 4 Medical Oncology, Hacettepe University Medical School, Ankara, TUR

**Keywords:** brain srs, gbm radiotherapy, lattice therapy, radiotherapy (rt), zap-x radiosurgical system

## Abstract

Lattice radiotherapy (LRT), a modern form of spatially fractionated radiation therapy, introduces a novel therapeutic strategy by delivering high-dose vertices interspersed within the gross tumor volume (GTV), leveraging both direct cytotoxicity and immune-mediated effects. While this technique has been explored in extracranial malignancies, its clinical application in intracranial tumors remains scarce. This study presents a preliminary case series assessing the feasibility, safety, and early clinical outcomes of LRT delivered via the gyroscopic radiosurgery platform ZAP-X® (ZAP Surgical Systems, Inc., San Carlos, USA) in patients with recurrent glioblastoma multiforme (GBM) who had already undergone multiple courses of radiotherapy.

Between December 2024 and February 2025, four patients with histologically confirmed GBM received single-fraction LRT with a prescribed dose of 20 Gy to each lattice vertex. Patient-specific lattice geometries were generated using a custom Python-based software tool, ensuring high-dose spheres were fully confined within the GTV and spaced to achieve a peak-to-valley dose ratio of 4. Treatment plans were created on the ZAP-X system using multi-isocenter targeting and optimized to deliver a mean GTV dose exceeding 5 Gy. The tumor volumes of the four presented cases were 186 cc, 68 cc, 75 cc, and 293 cc. Treatment delivery times ranged between 44 and 46 minutes. All patients were evaluated clinically and radiologically at 1 and 3 months post-treatment.

LRT was successfully delivered in all cases with favorable treatment tolerability and no acute neurotoxicity. Early imaging revealed radiological response or disease stabilization in three patients. One patient demonstrated significant cystic transformation and maintained stable neurological status; another exhibited partial response prior to disease progression at a distant intracranial site. The third patient remained clinically and radiologically stable without requiring systemic therapy. The fourth patient, who had a poor baseline status, experienced local progression and was hospitalized due to aspiration pneumonia. Across the cohort, steroid requirements were stable or reduced post-treatment.

These early findings suggest that ZAP-X-based LRT is a feasible and well-tolerated reirradiation option in heavily pretreated GBM patients. By integrating high-dose and low-dose regions within the tumor, LRT may stimulate immunologic effects and improve local control without increasing toxicity. Although limited by small sample size and short follow-up, this study supports the potential of LRT as a palliative strategy and sets the foundation for future prospective trials exploring its clinical efficacy and immunomodulatory role in neuro-oncology.

## Introduction

Spatially Fractionated Radiation Therapy (SFRT) is a non-conventional radiotherapy (RT) approach characterized by the deliberate delivery of non-uniform dose distributions within the gross tumor volume (GTV) [1, 2]. Unlike conventional external beam RT, which aims to deliver a homogeneous dose throughout the target volume, SFRT introduces regions of high-dose "peaks" interspersed with low-dose "valleys" within the tumor [3]. This technique exploits the spatial heterogeneity in radiation sensitivity across the tumor and its microenvironment.

The radiobiological basis of SFRT stems from several hypothesized mechanisms [4, 5]. High-dose regions can induce vascular damage, modulate hypoxia, and enhance pro-inflammatory cytokine release, thereby promoting indirect tumor cell kill. Sublethal irradiation in valley regions may induce cell death through immune-mediated bystander effects or systemic immune responses, contributing to tumor control beyond the directly irradiated areas. The preservation of intervening normal tissue between high-dose regions allows for enhanced repair of sublethal damage, thereby potentially increasing the therapeutic ratio.

SFRT can be implemented using various modalities. Lattice Radiotherapy (LRT) is a modern 3D adaptation of SFRT in which high-dose vertices (3D spherical or cylindrical volumes) are placed within the tumor, often spaced geometrically or based on biological imaging criteria (e.g., hypoxia, proliferation markers) [6-9]. Planning is typically performed using advanced treatment planning systems and delivered with intensity-modulated radiation therapy (IMRT), volumetric-modulated arc therapy (VMAT), or stereotactic techniques [10, 11].

LRT has been primarily explored in the management of large or bulky tumors, such as soft tissue sarcomas, head and neck cancers, and pelvic malignancies, where conventional uniform-dose radiation therapy often presents limitations due to normal tissue constraints. In contrast, the application of LRT in intracranial neoplasms remains relatively limited, largely due to the typically smaller size of these lesions. However, in the context of recurrent glioblastoma (GBM), a highly aggressive and radioresistant tumor that has undergone multiple lines of treatment with limited response to conventional fractionation schemes, LRT presents a promising therapeutic strategy by enabling the delivery of spatially intensified doses to selected tumor sub-volumes while sparing adjacent healthy tissue. LRT's main advantage in reirradiation settings lies in its ability to deliver highly conformal and selective doses, a necessity when cumulative dose constraints limit exposure to previously irradiated normal brain tissue.

In this context, we hypothesize that the gyroscopic design and dosimetric advantages of the ZAP-X® (ZAP Surgical Systems, Inc., San Carlos, USA) system may enable the feasible and effective delivery of LRT in the reirradiation of patients with glioblastoma (GBM). This approach has the potential to enhance tumor control while minimizing normal tissue toxicity, particularly in previously irradiated brain regions where conventional reirradiation strategies are limited.

## Case presentation

Patients and follow-up

GBM patients referred to our department for reirradiation were considered eligible for LRT. The clinical implementation of LRT using the ZAP-X system commenced in December 2024. Since then, eight patients have been treated with this technique.

Follow-up assessments were scheduled at 1, 3, and 6 months post-treatment and included magnetic resonance imaging (MRI) as well as clinical evaluations. Additional information was obtained from the institutional electronic medical record system.

For the present study, four patients were included based on the availability of serial post-treatment MRIs suitable for evaluation. These cases represent the initial cohort with sufficient follow-up imaging data for preliminary analysis of treatment response and feasibility.

Pre-treatment imaging and segmentation

All patients underwent both computed tomography (CT) and MRI prior to the planning of LRT. CT simulations were performed in the treatment position using appropriate immobilization devices. Subsequently, rigid image registration was conducted between the CT and MRI datasets, with particular emphasis on T2-weighted fluid-attenuated inversion recovery (T2-FLAIR) sequences to enhance the visualization of tumor margins.

Gross tumor volumes (GTVs) were delineated on the planning CT images under the guidance of co-registered T2-FLAIR MR images, ensuring accurate anatomical localization.

To define and position the lattice vertices within the GTV, we developed a custom Python-based software tool. This program enables automated placement of spherical high-dose vertices within the GTV based on user-defined geometric constraints. Specifically, the tool allows the user to set: the minimum distance between the lattice spheres and the GTV boundary, the radius of each lattice sphere, and the center-to-center spacing between adjacent spheres.

For this study, these parameters were defined as 5 mm (minimum margin to GTV boundary), 10 mm (sphere radius), and 30 mm (center-to-center spacing), respectively. The algorithm ensured that only spheres fully enclosed within the GTV were selected for dose boosting, thereby minimizing unintended dose spillage to adjacent normal brain tissue.

Lattice radiotherapy planning

**Figure 1:**
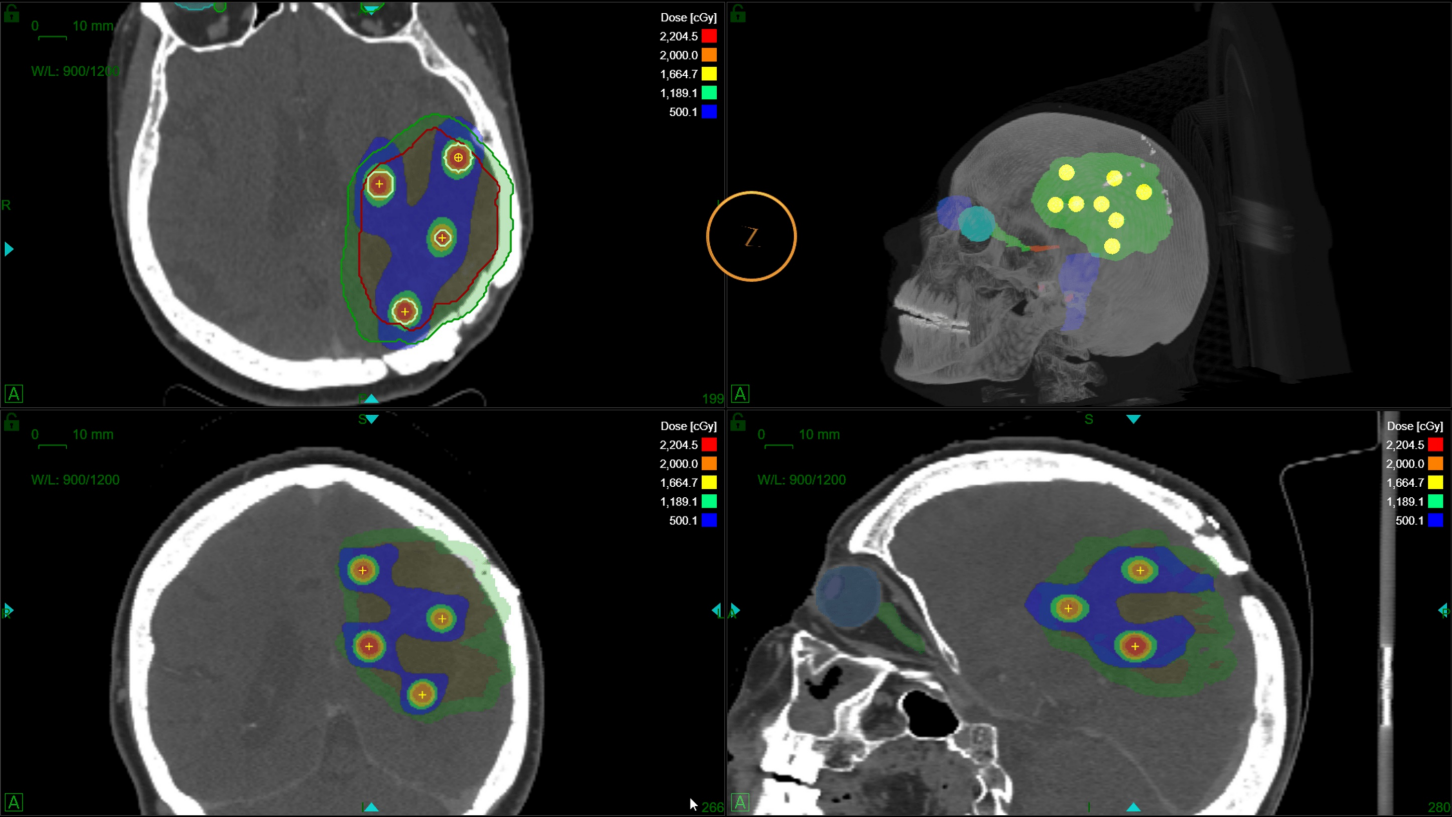
Example of lattice sphere placement and corresponding isocenter distribution for Case 1. Spheres were systematically placed within the gross tumor volume (GTV) according to predefined geometric criteria, ensuring minimal overlap and controlled out-of-volume extension. The resulting isocenter locations illustrate the spatial distribution intended for lattice radiotherapy planning.

Treatment planning was conducted using the novel gyroscopic radiosurgery system ZAP-X, which offers high geometric precision and steep dose gradients due to its unique design [12]. Multi-isocenter treatment plans were generated, with each lattice sphere being targeted using a 10 mm circular collimator to deliver highly conformal dose distributions. An example of lattice sphere placement and corresponding isocenter distribution for Case 1 is illustrated in Figure 1.

The prescribed dose was 20 Gy in a single fraction to each lattice vertex. Treatment plans were optimized to achieve a peak-to-valley dose ratio (PVDR) of 4, ensuring adequate dose fall-off between high-dose vertices and surrounding tissue. Additionally, a planning constraint was applied such that the mean dose to the entire GTV exceeded 5 Gy, thereby maintaining a therapeutic dose level across the tumor volume while leveraging the spatial fractionation benefits of the lattice approach. Table 1 presents an overview of the treatment parameters for the included cases. 

**Table 1:** Treatment Parameters of the Patients Abbreviations: GTV = gross tumor volume, CI = conformity index, HI = homogeneity index, GI = gradient index, V = volume.

	GTV (cc)	Mean GTV dose (cGy)	Maximum lattice dose (cGy)	Minimum lattice dose (cGy)	CI	HI	GI	Lattice volume (cc)	V_vertice_/V_GTV_(%)	Vertex number
Case 1	186	910	2370	1865	1.04	1.2	7.1	4.1	2.2	8
Case 2	68	740	2228	1914	1.21	1.11	6.8	3.1	4.5	6
Case 3	75	780	2315	1790	1.15	1.21	7.7	3.6	4.8	7
Case 4	293	758	2478	1954	1.05	1.14	5.4	6.2	2.1	12

Case 1

A 70-year-old female patient was diagnosed with an intracranial tumor in the left parietal lobe in 2017. Following gross total resection, pathological analysis confirmed GBM, isocitrate dehydrogenase (IDH) wild type, grade IV, according to the 2016 World Health Organization (WHO) Classification of Tumors of the Central Nervous System. She subsequently received adjuvant RT, a total dose of 60 Gy in 30 fractions, concomitant with temozolomide (75 mg/m²). After completing chemoradiation (CRT), she underwent 16 cycles of adjuvant temozolomide.

In September 2020, she presented to the emergency department with right arm numbness and speech difficulties. MRI revealed recurrence in the left parietal lobe within the initial radiation field. After multidisciplinary evaluation, surgery was recommended, but the patient declined. She was instead offered reirradiation and received 30 Gy in five fractions to the recurrent lesion, followed by temozolomide. Initial follow-up imaging showed tumor regression; however, at the six-month MRI, suspicion of progression was noted. The lesion was subsequently excised, and pathology confirmed radiation necrosis. The patient remained under surveillance with MRI scans every three months until November 2024, when imaging revealed a solid contrast-enhancing mass with cystic components, measuring 6.1 × 4.4 cm in the largest axial dimension, within the prior radiation field, indicating progressive disease. She also exhibited neurological deterioration, and the multidisciplinary tumor board recommended reirradiation. In December 2024, she underwent LRT with a single fraction of 20 Gy using ZAP-X gyroscopic radiosurgery, following the technique previously described. The tumor volume was 186 cc. The procedure lasted 46 minutes, after which the patient was prescribed 8 mg dexamethasone and antiemetics, with temozolomide initiated subsequently.

**Figure 2:**
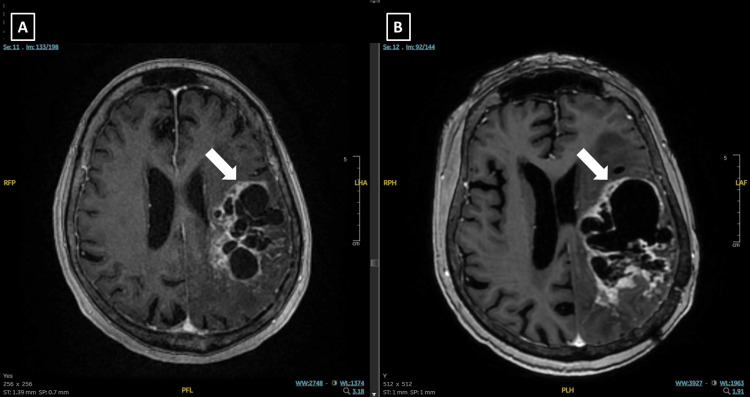
Diagnostic and 3-month follow-up MRI images of Case 1. (A) Axial T1-weighted post-contrast (T1C+) MRI at diagnosis showing a solid contrast-enhancing mass with cystic components in the left temporal lobe, measuring 6.1 × 4.4 cm. (B) Axial MRI at the 3-month follow-up after LATTICE radiotherapy. The lesion shows an increase in cystic components, with persistent T2 hyperintensity and more prominent contrast enhancement compared to the 1-month post-treatment scan.

At the one-month follow-up MRI, the solid tumor components had regressed. At three months post-treatment, MRI demonstrated an increase in the cystic component of the lesion, with stable T2 hyperintense changes compared to the one-month scan and more pronounced contrast enhancement (Figure 2). Despite radiological findings suggestive of progression, the patient remained neurologically stable without new deficits but experienced occasional episodes of confusion. A 4 mg increase in steroid dosage was recommended, and she continues to be closely monitored.

Case 2

The second patient was a 50-year-old male who presented with right extremity numbness in June 2021. MRI revealed a high-grade glial tumor in the left frontal lobe. He underwent gross total resection, and histopathological analysis confirmed GBM, WHO grade IV. He received adjuvant CRT with a total dose of 60 Gy in 30 fractions, concurrently with temozolomide, followed by 20 cycles of adjuvant temozolomide.

After a 13-month progression-free interval, MRI demonstrated a new expansile infiltrative lesion in the contralateral hemisphere, localized to the right parietal lobe. In May 2024, surgical resection was performed, and histopathology confirmed GBM, IDH wild type, WHO grade IV. The lesion was evaluated as a second primary tumor, and CRT was administered with a total dose of 60 Gy in 30 fractions with concurrent temozolomide, followed by five cycles of adjuvant temozolomide.

After five months of follow-up, MRI demonstrated an increase in the size of contrast-enhancing lesions within the second radiation field, consistent with disease progression. Due to the infiltrative nature of the tumor in eloquent areas, the multidisciplinary tumor board did not recommend surgical intervention. Instead, reirradiation with a total dose of 20 Gy using the LRT was performed in January 2025. The tumor volume was 68 cc, and the treatment duration was 44 minutes.

**Figure 3:**
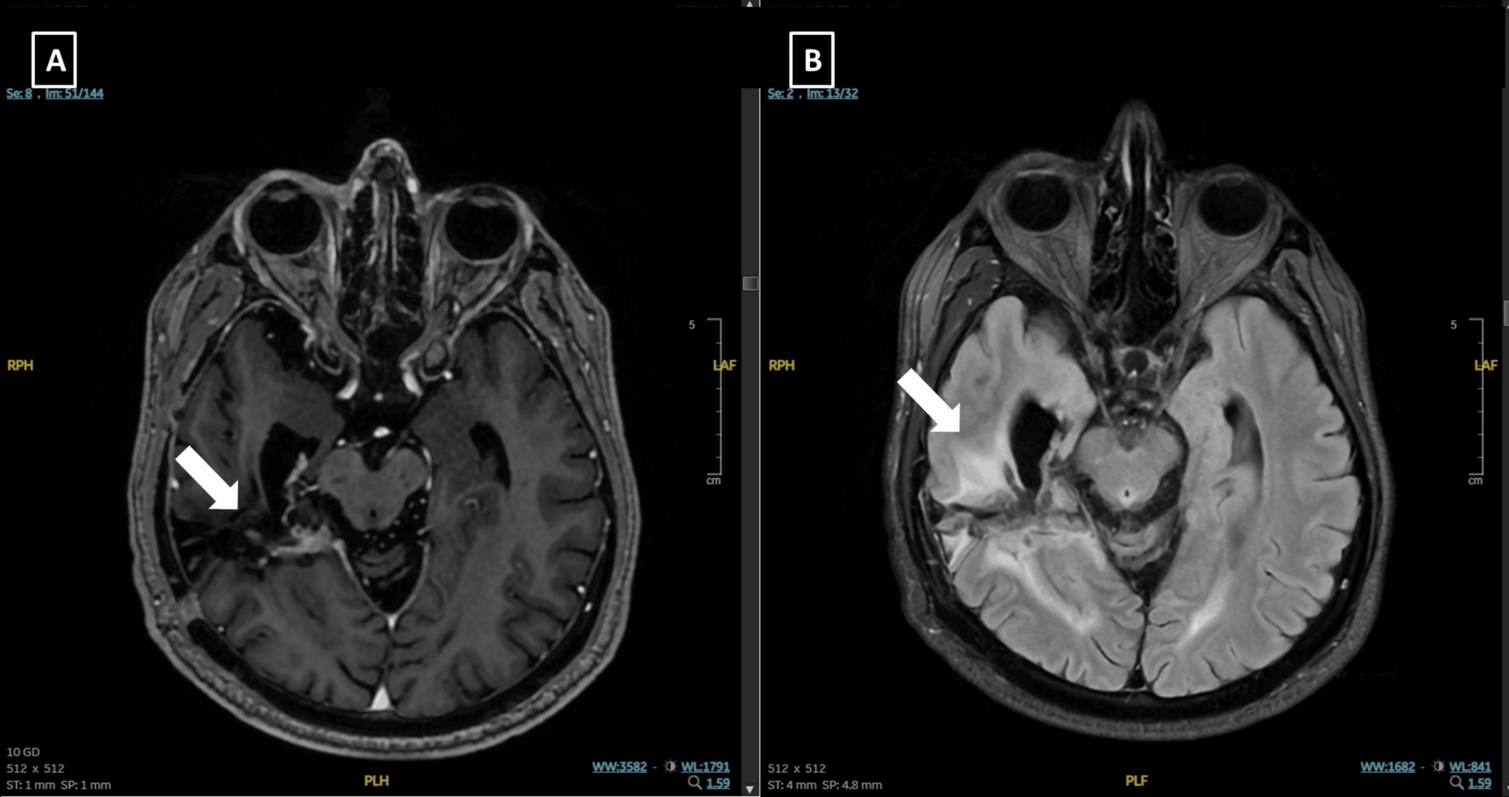
Diagnostic MRI images of Case 2. (A) Axial T1-weighted post-contrast (T1C+) MRI demonstrating a recurrence involving the left frontal and parietal lobes. (B) Axial T2 FLAIR image corresponding to the lesion.

At the one-month follow-up MRI, significant regression was observed in contrast-enhancing areas and regions of enhanced T2 intensity (Figure 3). Following reirradiation, the patient received bevacizumab, and steroids were tapered. However, at the last follow-up in March 2025, MRI revealed a new expansile lesion at the left superior cerebellar region, causing mild narrowing of the fourth ventricle, consistent with high-grade glioma progression. The patient was subsequently admitted to the intensive care unit due to impaired oral intake and confusion and later succumbed to disease progression.

Case 3

The third patient was a 64-year-old woman who presented in February 2017 with headache and confusion. Imaging revealed a high-grade glial tumor in the left temporoparietal region. She underwent subtotal resection, and pathological analysis confirmed GBM. Adjuvant chemoradiotherapy was administered to a total dose of 60 Gy in 30 fractions, followed by 26 cycles of adjuvant temozolomide. At our institution, continuation of adjuvant temozolomide beyond the standard six cycles may be considered on a case-by-case basis, taking into account factors such as a favorable clinical course, lack of radiological progression, and minimal treatment-related toxicity.

**Figure 4:**
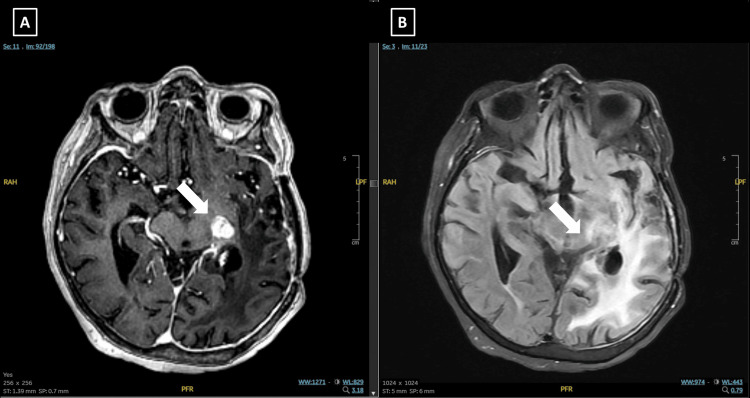
Diagnostic MRI images of Case 3. (A) Axial T1-weighted post-contrast (T1C+) MRI demonstrating a recurrence involving the left frontal and parietal lobes. (B) Axial T2 fluid-attenuated inversion recovery (FLAIR) image corresponding to the lesion.

She remained progression-free for 80 months, until an MRI revealed a recurrent lesion within the initial radiation field. After evaluation by a multidisciplinary tumor board, she underwent subtotal resection, and pathology once again confirmed GBM. Following surgery, she was treated with reirradiation at a dose of 30 Gy in five fractions, and temozolomide was reinitiated. After five cycles of temozolomide, new contrast-enhancing lesions suspicious for recurrence or radiation necrosis were detected on MRI, accompanied by deteriorating neurological symptoms. A second surgical intervention was performed, and pathology revealed radiation necrosis. Bevacizumab was subsequently initiated. Following nine cycles of bevacizumab, MRI revealed a new recurrence involving the left frontal and parietal lobes. Surgical resection was not considered feasible, and reirradiation with LRT was performed in February 2025 (Figure 4). The tumor volume was 75 cc, and the treatment duration was 46 minutes.

She is currently doing well without new neurological symptoms. Steroids were successfully tapered. Post-radiation, she has been evaluated by the medical oncology team, but no additional systemic therapy has been initiated to date.

Case 4

The fourth patient was a 68-year-old female who presented to the neurology outpatient clinic in September 2021 with a speech disorder. MRI revealed a mass in the left temporal lobe consistent with a high-grade glial tumor. She underwent subtotal resection, and pathology confirmed gliosarcoma. Adjuvant chemoradiotherapy was administered with a total dose of 60 Gy in 30 fractions alongside concurrent temozolomide, followed by 12 cycles of adjuvant temozolomide. In January 2023, follow-up MRI revealed a recurrence at the resection cavity. Surgery was performed based on multidisciplinary tumor board recommendation, and pathology confirmed GBM, IDH wild type. Postoperative MRI revealed residual disease, and the patient received stereotactic radiotherapy (SRT) with a total dose of 30 Gy in five fractions to the resection cavity and residual mass. She was subsequently treated with irinotecan and bevacizumab as second-line chemotherapy.

After a one-year progression-free interval, MRI revealed a non-contrast-enhancing lesion with diffusion restriction adjacent to the operative cavity, consistent with recurrence. The patient was re-evaluated by the tumor board, and surgery was again recommended. Pathology confirmed recurrent GBM. Following the procedure, she developed a prolonged wound infection and was diagnosed with osteomyelitis, requiring decompressive craniotomy and intravenous antibiotic therapy. Temozolomide was restarted; however, four months later, progression was observed in the left anterior temporal lobe, basal ganglia, and posterior insular region. A third course of radiotherapy was planned, and LRT was delivered. The tumor volume was 293 cc, and treatment lasted 44 minutes. The patient received 8 mg of dexamethasone and antiemetics prior to treatment. Due to her poor performance status, systemic treatment was not initiated.

**Figure 5:**
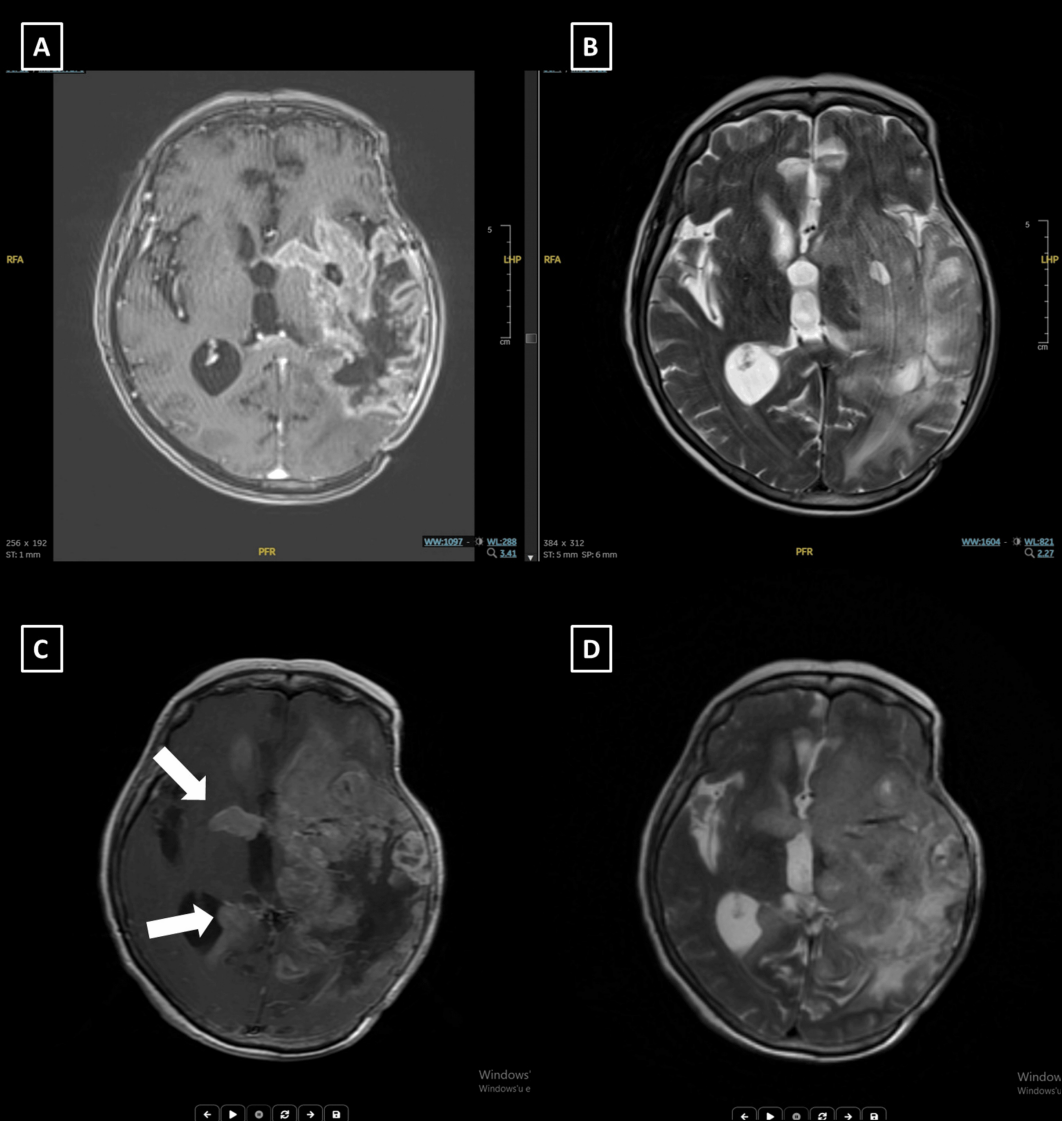
Diagnostic and 3-month follow-up MRI images of Case 4. (A, B) Diagnostic MRI: Axial T1-weighted post-contrast (T1C+) (A) and T2 fluid-attenuated inversion recovery (FLAIR) (B) images showing a recurrent lesion involving the left anterior temporal lobe, basal ganglia, and posterior insular region. (C, D) Three-month follow-up MRI after LATTİCE radiotherapy: Axial T1C+ (C) and T2 FLAIR (D) images demonstrate lesion progression with extension into the contralateral hemisphere.

At the time, she had swallowing difficulties and was dependent on a nasogastric tube. She remained clinically stable following LRT but was later hospitalized for aspiration pneumonia. Follow-up MRI demonstrated disease progression with extension to the contralateral hemisphere (Figure 5).

## Discussion

The present study reports the initial clinical experience with LRT using the ZAP-X system for re-reirradiation in patients with recurrent GBM. To our knowledge, this is one of the first series to describe the feasibility and early outcomes of LRT delivered via a gyroscopic radiosurgery platform in the pretreated GBM population. Our cohort consisted of four patients, each with a history of at least two prior courses of radiotherapy and substantial disease burden (Table 2).

**Table 2:** Clinical Characteristics, Treatment Details, and Outcomes of Patients Undergoing LRT with ZAP-X Radiosurgery Abbreviations: RT = radiotherapy, LRT = Lattice Radiotherapy, F = female, M = male, fx = fraction, AWD = alive with disease, EWD = exitus with disease.

Case	Age/Sex	Tumor Location	Prior RT Dose/Fractions	Reirradiation Dose/Fractions (LRT)	Treatment Duration (min)	Response (at 1 mo)	Progression / Follow-up	Toxicity & Steroid Use	Outcome/Status
1	70F	Left parietal	60 Gy / 30 fx	20 Gy / 1 fx	46	Tumor regression	Cystic progression at 3 mo; Neurologically stable with increased steroids	No acute neurotoxicity; steroids increased	AWD
2	50M	Left frontal & right parietal (second tumor)	60 Gy / 30 fx (x2)	20 Gy / 1 fx	44	Marked radiologic response	Distant progression (cerebellar) at 3 mo; deceased	No acute neurotoxicity; steroids tapered	EWD
3	64F	Left temporoparietal	60 Gy / 30 fx	20 Gy / 1 fx	46	Stable / no new symptoms	No progression at last follow-up	Steroids tapered successfully	AWD
4	68F	Left temporal (gliosarcoma)	60 Gy / 30 fx	20 Gy / 1 fx	44	Clinically stable post-LRT	Progression in contralateral hemisphere	Aspiration pneumonia (systemic); steroids maintained	AWD

All third-course irradiations were classified as Type 1 reirradiation according to the European Society for Radiotherapy and Oncology (ESTRO) and European Organization for Research and Treatment of Cancer (EORTC) consensus guidelines on reirradiation [13]. Despite these adverse prognostic factors, LRT was technically feasible in all cases, with acceptable treatment times (approximately 44 minutes) and adequate plan quality metrics, including a peak-to-valley dose ratio (PVDR) of 4 and mean GTV dose exceeding 5 Gy. These planning characteristics are consistent with prior reports of LRT in extracranial malignancies and support the adaptability of the technique to intracranial tumors [14-16].

Reirradiation in GBM remains a therapeutic challenge due to the cumulative radiation dose to normal brain tissue and the inherent radioresistance of recurrent tumors [17-19]. In recent years, LRT has emerged as a novel SFRT technique that aims to deliver high-dose “vertices” within the GTV, while maintaining a lower “valley” dose in surrounding tissues. This approach is hypothesized to elicit both direct cytotoxic and immune-mediated effects, thereby enhancing tumor control while mitigating toxicity.

In the immunotherapy era, the immune-mediated effects of LRT are particularly noteworthy. The immunomodulatory potential of RT has been demonstrated across various dose and fractionation schedules. Low-dose RT (≤2 Gy) has been shown to reprogram the tumor microenvironment by enhancing infiltration of tumor-infiltrating lymphocytes (TILs) and natural killer (NK) cells, promoting antitumor immunity through M1 macrophage polarization, and reducing levels of the immunosuppressive cytokine tumor growth factor-beta (TGF-β)[20, 21]. On the other hand, ablative doses of RT induce immunogenic cell death, increase the presence of antigen-presenting cells, and facilitate greater immune cell infiltration into the tumor [22]. Additionally, high-dose RT leads to vascular disruption, triggering indirect cell death mechanisms [23]. LRT uniquely capitalizes on the synergistic immunologic benefits of both low- and high-dose RT by integrating them within a single treatment session. Clinical studies further support the complementary nature of these dose ranges. In a phase II study by Patel et al. [24], the combination of high- and low-dose RT was used to enhance lesion-specific responses in patients with immunotherapy-resistant solid tumors, with low-dose RT specifically augmenting immune cell infiltration into the tumor microenvironment.

Upon closer examination, early post-treatment imaging showed tumor regression or clinical stabilization in three of the four patients (Table 2). Notably, Case 1 demonstrated cystic transformation and possible radiological progression but maintained stable neurological status at three months, while Case 2 exhibited a marked radiological response before eventual progression in a distant brain region. Furthermore, the prolonged disease stability observed in Case 3, despite prior progression after multiple therapies, highlights the potential of LRT as a palliative yet meaningful treatment option for selected patients. Treatment-related toxicity was generally manageable across the cohort; steroid requirements remained stable or decreased in most cases, and no acute neurotoxicity attributable to LRT was observed. However, systemic complications such as aspiration pneumonia (Case 4) and decline due to extracranial complications emphasize the importance of careful patient selection and multidisciplinary management. Additionally, concomitant therapies may further enhance therapeutic efficacy.

Initial immunotherapy trials in recurrent glioblastoma have yielded disappointing results, and immunotherapy is currently not recommended outside the context of clinical trials [25-28]. For instance, the CheckMate 143 trial demonstrated that nivolumab did not offer a survival advantage over bevacizumab in patients with recurrent GBM [28]. These outcomes are largely attributed to the intrinsic heterogeneity of GBM, its low tumor mutational burden, and the profoundly immunosuppressive tumor microenvironment [29]. Strategies aimed at modulating the tumor microenvironment, such as LRT, may hold potential in overcoming these barriers and enhancing the efficacy of future immunotherapeutic approaches.

In the setting of reirradiation, dose constraints to surrounding critical structures pose a significant limitation-particularly in cranial treatments where organ-at-risk (OAR) tolerance is low. In such scenarios, LRT offers a potential solution by enabling spatial dose modulation, thereby delivering high-dose regions within the tumor while sparing adjacent normal tissues. While the efficacy of LRT has been previously demonstrated in extracranial tumors, its application in intracranial lesions remains limited. One contributing factor may be the technical constraints of conventional treatment systems. For instance, Ertan et al. [30] explored the feasibility of intracranial LRT using the CyberKnife system (Accuray, Madison, USA) but reported treatment durations of approximately 3 to 4 hours, which may not be clinically acceptable due to patient comfort and workflow limitations. While this demonstrates technical feasibility, such prolonged treatment times may pose practical challenges in terms of patient comfort and clinical workflow efficiency. This situation may be related to the use of an earlier version of the CyberKnife system in the aforementioned study; newer versions incorporating multileaf collimator (MLC) systems may enable shorter treatment times.

The ZAP-X gyroscopic radiosurgery system has shown the potential for reduced treatment durations in our experience, which may offer workflow advantages in selected cases. In our clinical experience, total treatment times under an hour, making single-fraction LRT for intracranial tumors both technically and logistically feasible. These results suggest that ZAP-X may be particularly well-suited for delivering spatially fractionated high-dose therapy in the brain, even in previously irradiated regions. Moreover, the ZAP-X system also offers unique advantages for delivering LRT. Its gyroscopic beam delivery and steep dose gradients facilitate the precise targeting of lattice spheres within complex intracranial geometries [12]. Our custom-developed Python-based planning tool further optimized vertex placement, ensuring adherence to spatial constraints and minimizing dose spillage. Future integration of this tool into treatment planning systems could streamline clinical workflows and enhance reproducibility. 

Despite promising early outcomes, limitations must be acknowledged. The small sample size of the study limits generalizability. In addition, the short follow-up duration precludes definitive conclusions regarding long-term efficacy or durability of response.

## Conclusions

LRT delivered with the ZAP-X system appears to be a technically feasible and well-tolerated approach for patients with recurrent GBM who have exhausted standard treatment options. The high geometric precision and steep dose gradients of ZAP-X enable selective dose escalation within the tumor while effectively sparing surrounding healthy brain tissue. Its short treatment duration and integrated planning workflow make ZAP-X a practical platform for implementing LRT in clinical settings. Early observations are encouraging, and further prospective studies are needed to validate its efficacy and identify optimal patient selection criteria.

## References

[1] Iori F, Cappelli A, D'Angelo E (2023). Lattice radiation therapy in clinical practice: a systematic review. Clin Transl Radiat Oncol.

[2] Iori F, Trojani V, Zamagni A, Ciammella P, Iori M, Botti A, Iotti C (2025). Spatially fractionated radiation therapy for palliation in patients with large cancers: a retrospective study. Adv Radiat Oncol.

[3] Li H, Mayr NA, Griffin RJ (2024). Overview and recommendations for prospective multi-institutional spatially fractionated radiation therapy clinical trials. Int J Radiat Oncol Biol Phys.

[4] Misa J, St Clair W, Pokhrel D (2025). Radiobiological modeling of indirect cell-kill mechanisms in spatially fractionated radiation therapy (SFRT) treatment of large and bulky unresectable tumors. Cureus J Med Sci.

[5] Rivera JN, Laemont K, Tovmasyan A (2025). Minibeam spatially-fractionated radiation therapy is superior to uniform dose radiation therapy for abscopal effect when combined with PD-L1 checkpoint inhibitor immunotherapy in a dual tumor murine mammary carcinoma model. Radiation.

[6] Zhu L, Ammar A, Chen Q, Rong Y (2025). Rapid VMAT LATTICE therapy planning using deep learning predicted synthetic CT from diagnostic CT scans. Cureus J Med Sci.

[7] Gaudreault M, Chang D, Kron T, Siva S, Chander S, Hardcastle N, Yeo A (2024). Development of an automated treatment planning approach for lattice radiation therapy. Med Phys.

[8] Gaudreault M, Yu KK, Chang D, Kron T, Hardcastle N, Chander S, Yeo A (2024). Automated lattice radiation therapy treatment planning personalised to tumour size and shape. Phys Med.

[9] Zhang W, Lin Y, Wang F, Badkul R, Chen RC, Gao H (2023). Lattice position optimization for LATTICE therapy. Med Phys.

[10] Mansfield R, Ginn J, Mullikin T (2025). Fully automating lung cancer LATTICE radiation therapy (LRT) using multi-focal dynamic conformal arc (DCA) delivery: a feasibility study. Cureus Journal of Medical Science.

[11] Wu X, Perez NC, Zheng Y (2020). The technical and clinical implementation of LATTICE radiation therapy (LRT). Radiat Res.

[12] Wang J, Zheng Q, Wang Y (2024). Dosimetric comparison of ZAP-X, Gamma Knife, and CyberKnife stereotactic radiosurgery for single brain metastasis. BMC Cancer.

[13] Andratschke N, Willmann J, Appelt AL (2022). European Society for Radiotherapy and Oncology and European Organisation for Research and Treatment of Cancer consensus on re-irradiation: definition, reporting, and clinical decision making. Lancet Oncol.

[14] At B, Velayudham R (2024). Assessing dosimetric advancements in spatially fractionated radiotherapy: from grids to lattices. Med Dosim.

[15] Prado A, Martí J, García de Acilu P (2024). Dosimetrical and geometrical parameters in single-fraction lattice radiotherapy for the treatment of bulky tumors: Insights from initial clinical experience. Phys Med.

[16] Zhang H, Wu X, Zhang X (2020). Photon GRID radiation therapy: a physics and dosimetry white paper from the Radiosurgery Society (RSS) GRID/LATTICE, Microbeam and FLASH Radiotherapy Working Group. Radiat Res.

[17] Amichetti M, Amelio D (2011). A review of the role of re-irradiation in recurrent high-grade glioma (HGG). Cancers (Basel).

[18] Zwirner K, Paulsen F, Schittenhelm J, Borchers C, Skardelly M, Zips D, Eckert F (2017). Prognostic parameters and outcome after re-irradiation for progressive glioblastoma. Acta Neurol Scand.

[19] García-Cabezas S, Rivin Del Campo E, Solivera-Vela J, Palacios-Eito A (2021). Re-irradiation for high-grade gliomas: Has anything changed?. World J Clin Oncol.

[20] Herrera FG, Ronet C, Ochoa de Olza M (2022). Low-dose radiotherapy reverses tumor immune desertification and resistance to immunotherapy. Cancer Discov.

[21] Barsoumian HB, Ramapriyan R, Younes AI (2020). Low-dose radiation treatment enhances systemic antitumor immune responses by overcoming the inhibitory stroma. J Immunother Cancer.

[22] Jarosz-Biej M, Smolarczyk R, Cichoń T, Kułach N (2019). Tumor microenvironment as a "game changer" in cancer radiotherapy. Int J Mol Sci.

[23] Rodríguez-Barbeito P, Díaz-Botana P, Gago-Arias A (2019). A model of indirect cell death caused by tumor vascular damage after high-dose radiotherapy. Cancer Res.

[24] Patel RR, He K, Barsoumian HB (2021). High-dose irradiation in combination with non-ablative low-dose radiation to treat metastatic disease after progression on immunotherapy: Results of a phase II trial. Radiother Oncol.

[25] Blumenthal DT, Yalon M, Vainer GW (2016). Pembrolizumab: first experience with recurrent primary central nervous system (CNS) tumors. J Neurooncol.

[26] Kurz SC, Cabrera LP, Hastie D (2018). PD-1 inhibition has only limited clinical benefit in patients with recurrent high-grade glioma. Neurology.

[27] Reardon DA, Kim TM, Frenel JS (2021). Treatment with pembrolizumab in programmed death ligand 1-positive recurrent glioblastoma: results from the multicohort phase 1 KEYNOTE-028 trial. Cancer.

[28] Reardon DA, Brandes AA, Omuro A (2020). Effect of nivolumab vs bevacizumab in patients with recurrent glioblastoma: The CheckMate 143 phase 3 randomized clinical trial. JAMA Oncol.

[29] Medikonda R, Dunn G, Rahman M, Fecci P, Lim M (2021). A review of glioblastoma immunotherapy. J Neurooncol.

[30] Ertan F, Yeginer M, Zorlu F (2023). Dosimetric performance evaluation of MLC-based and cone-based 3D spatially fractionated LATTICE radiotherapy. Radiat Res.

